# Supplementary material for: Analysis of proposed carbon capture projects in the US power sector and co-location with environmental justice communities
Source: PLoS One. 2025 May 16;20(5):e0323817. doi: 10.1371/journal.pone.0323817 (PMC12084031; doi:10.1371/journal.pone.0323817)
Supplement: S1 Table — (PDF) [file pone.0323817.s001.pdf]

S1 Table shows the databases used for this analysis, as well as additional databases that were not included but could be used for future analyses.

S1 Table. CCS Databases

CCS Database	Used in Analysis?	Access Date	Link
US Department of Energy National Energy Technology Laboratory (DOE NETL) CCS Database	Yes	April 11, 2023	https://netl.doe.gov/carbon-management/carbon-storage/worldwide-ccs-database
Global CCS Institute Facilities Database	Yes	May 2, 2023	https://co2re.co/FacilityData
International Energy Agency (IEA) CCUS Projects Database	Yes	May 2, 2023	https://www.iea.org/data-and-statistics/data-product/ccus-projects-database
Clean Air Task Force (CATF) US Carbon Capture Project Table	Yes	May 2, 2023	https://www.catf.us/ccstableus/
Rhodium Group and MIT Center for Energy and Environmental Policy Research Clean Investment Monitor	No	N/A	https://rhg.com/research/clean-investment-monitor/
Cipher Cleantech Tracker	No	N/A	https://www.ciphernews.com/cleantech-tracker/
