# Supplementary material for: Analysis of proposed carbon capture projects in the US power sector and co-location with environmental justice communities
Source: PLoS One. 2025 May 16;20(5):e0323817. doi: 10.1371/journal.pone.0323817 (PMC12084031; doi:10.1371/journal.pone.0323817)
Supplement: S1 Appendix — (PDF) [file pone.0323817.s002.pdf]

S1 Appendix. List of proposed CCS facilities

- Broadwing Clean Energy Complex / Illinois Allam-Fetvedt cycle power plant (IL)
- CalCapture CCS+ Elk Hills power plant (CA)
- Calpine Baytown Energy Center (TX)
- Calpine Deer Park Energy Center (TX)
- Calpine Delta Energy Center (CA)
- Carbon Capture Retrofit at Sherman Generating Station (TX)
- CO2 capture at Coal Creek** (ND)
- CO2 capture at Duke Energy's East Bend Station (KY)
- Competitive Power Ventures CCGT CPV Shay Energy Center (WV)
- Coyote Clean Power Project (CO)
- Dry Fork Integrated Commercial CCS (WY)
- Fidelis Project Cyclus Baton Rouge (LA)
- Gerald Gentleman Station Carbon Capture** (NE)
- James M Barry Electric Generating Plant CCUS (AL)
- Kern River Eastridge cogeneration plant San Joaquin Valley (CA)
- LG&E Cane Run NGCC CR7 (KY)
- Mendota BECCS power project (Clean Energy Systems Carbon Negative Energy Plant - Central
- Valley) (CA)
- Mustang Station Carbon Capture (TX)
- Net Power Odessa gas plant (TX)
- Plant Daniel Carbon Capture (MS)
- Prairie State Generating Station Carbon Capture (IL)
- Project Tundra at Milton R Young Station (ND)
- Tampa Electric Company Polk Power station NGCC (FL)
- The ZEROS project Chambers & Liberty County (Jefferson) (TX)
- Clean Energy Systems Delano Bioenergy with Carbon Removal & Storage (BiCRS) Plant - Madera
- County (CA)
- Dave Johnston Plant Carbon Capture (WY)
- Diamond Vault CCS (LA)
- San Juan Generating Station Carbon Capture (NM)
- CWLP Carbon Capture Project (IL)
- Quail Run Carbon Capture Project (TX)
- Covanta (IN)
- Baytown Low Carbon Hydrogen Project (TX)
- Filer City Biomass CCS Project (MI)
- CarbonSAFE Eos (CO)
- Illinois Basin West CarbonSAFE (IL)

** Facilities that are not within three miles of an EJ community
